# Magnetic Rotation in the A = *80* Region: *M1* Bands in Heavy *Rb* Isotopes

**DOI:** 10.6028/jres.105.017

**Published:** 2000-02-01

**Authors:** R. Schwengner, H. Schnare, S. Frauendorf, F. Dönau, L. Käubler, H. Prade, E. Grosse, A. Jungclaus, K. P. Lieb, C. Lingk, S. Skoda, J. Eberth, G. de Angelis, A. Gadea, E. Farnea, D. R. Napoli, C. A. Ur, G. Lo Bianco

**Affiliations:** Institut für Kern- und Hadronenphysik, FZ Rossendorf, 01314 Dresden, Germany; II. Physikalisches Institut, Universität Göttingen, 37073 Göttingen, Germany; Institut für Kernphysik, Universität zu Köln, 50937 Köln, Germany; INFN, Laboratori Nazionali di Legnaro, 35020 Legnaro, Italy; INFN, Sezione di Milano, 20133 Milano, Italy

**Keywords:** in-beam γ-spectroscopy, magnetic dipole bands, tilted-axis cranking model

## Abstract

We have studied the isotopes ^82^Rb_45_, ^83^Rb_46_, and ^84^Rb_47_ to search for magnetic rotation which is predicted in the tilted-axis cranking model for a certain mass region around *A* = 80. Excited states in these nuclei were populated via the reaction ^11^B + ^76^Ge with *E* = 50 MeV at the XTU tandem accelerator of the LNL Legnaro. Based on a γ-coincidence experiment using the spectrometer GASP we have found magnetic dipole bands in each studied nuclide. The regular M1 bands observed in the odd-odd nuclei ^82^Rb and ^84^Rb include *B*(M1)/*B*(E2) ratios decreasing smoothly with increasing spin in a range of 13^−^ ≤ J^π^ ≤ 16^−^. These bands are interpreted in the tilted-axis cranking model on the basis of four-quasiparticle configurations of the type $\pi \left(fp\right)\;\pi g_{9/2}^{2}\;\nu g_{9/2}$. This is the first evidence of magnetic rotation in the *A* ≈ 80 region. In contrast, the M1 sequences in the odd-even nucleus ^83^Rb are not regular, and the *B*(M1)/*B*(E2) ratios show a pronounced staggering.

## 1. Introduction

In the tilted-axis cranking (TAC) model [1], which considers the rotation of the nucleus about axes tilted with respect to the principal axes, a new rotational mode referred to as magnetic rotation has been established. This mode is expected to appear in nuclei with small deformation, if multi-quasiparticle configurations are formed from high-*j* proton particles and high-*j* neutron holes or vice versa. The coupling of these configurations results in a large transverse magnetic moment. The rotating magnetic dipole gives rise to the emission of magnetic dipole (M1) radiation in contrast to the electric quadrupole (E2) radiation induced by the rotating deformed electric charge distribution in the case of conventional rotation. In the case of magnetic rotation the total spin is built up by the gradual alignment of the spins of the high-*j* nucleons (“shears mechanism”). This concept has been applied for the first time to the M1 bands (“shears bands”) discovered in nuclei around ^200^Pb [2]. The predicted decrease of the M1 transition strength with increasing spin caused by the gradual alignment of the individual spin vectors (closing of the shears) has recently been experimentally proven for the M1 bands in ^198,199^Pb [3]. Magnetic rotation is also predicted for other mass regions of the nuclear chart [4]. Indeed, it has recently been observed in ^105^Sn [5], ^110^Cd [6], and ^139^Sm [7].

Among the mass regions, where magnetic rotation is predicted to occur, there is also the region around *A* = 80 [4]. There, the particle-like protons fill successively the *fp* and the high-*j* intruder *g*_9/2_ levels while hole-like neutrons occupy the *g*_9/2_ level. Indeed, sequences of intense M1 transitions starting at about *E* ≈ 3 MeV have been found in several Br, Rb, and Kr isotopes (see, e.g., [8] and Refs. therein) but there is too little experimental information so far to prove the appearance of magnetic rotation. To search for experimental evidence of the predicted magnetic rotation in this region we have investigated the nuclides ^82^Rb_45_, ^83^Rb_46_, and ^84^Rb_47_.

## 2. Experimental Results

**Fig. 1:**
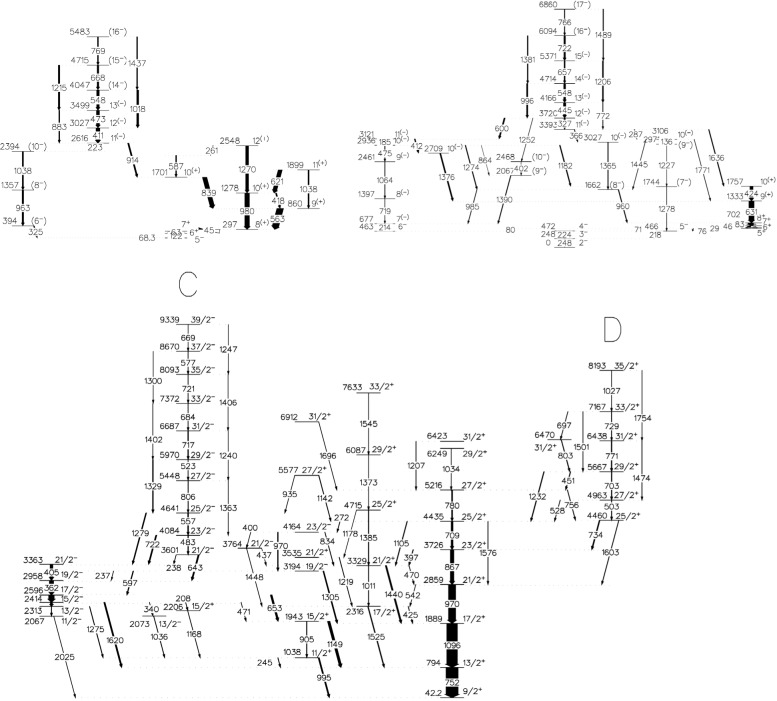
Partial level schemes of ^82^Rb (top left) and ^84^Rb (top right) and ^83^Rb (bottom) deduced from this work.

Excited states in ^82,83,84^Rb were populated via the reaction ^11^B + ^76^Ge at *E* = 50 MeV using the ^11^B beam of the XTU tandem accelerator of the LNL Legnaro. γ rays were detected with the spectrometer GASP. A total of 1.5 × 10^8^ three-fold coincidence events was recorded in a thin-target experiment. On the basis of this experiment we have found several new band structures with respect to previous work [9,10]. In particular, M1 bands have been found for the first time in each studied nuclide. Partial level schemes including these bands found in the present experiment are shown in Fig. 1. These level schemes result from γ-γ and γ-γ-γ coincidence relations and γ-ray intensities. Spin and parity assignments are based on γ-γ directional correlations and deexcitation modes.

## 3. Interpretation

**Fig. 2:**
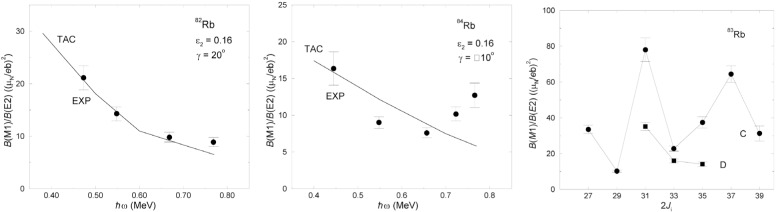
Experimental and calculated *B*(M1)/*B*(E2) ratios of the negative-parity M1 bands in ^82^Rb (left panel) and ^84^Rb (middle panel). Experimental *B*(M1)/*B*(E2) ratios of the M1 bands C and D in ^83^Rb (right panel).

The M1 bands of negative parity observed in the odd-odd nuclei ^82^Rb and ^84^Rb are regular (*E_γ_* ∝ J). The *B*(M1)/*B*(E2) ratios deduced from the intensities of transitions deexciting a certain state of the M1 band reach values up to 25 (μ_N_/eb)^2^ and decrease smoothly with increasing spin in a range of 13 ≤ J ≤ 16. This is an important characteristic of magnetic rotation. Thus, we have interpreted these bands in the framework of the TAC model [1]. In the calculations, the lowest-lying four-quasiparticle (4*qp*) configuration for *Z* = 37 and *N* = 45, 47 turns out to be $\pi \left(fp\right)\;\pi g_{9/2}^{2}\;\nu g_{9/2}$, which has been adopted. The parameter *κ* of the QQ interaction was adjusted such that in a calculation for the even-even neighbor 82Kr the experimental *B*(E2, 2^+^ → 0^+^) [11] value is reproduced and in the case of ^84^Rb scaled according to *κ* ∝ *A*^−5/3^. An equilibrium deformation of *ϵ*_2_ = 0.16 was obtained for the adopted 4*qp* configuration in both ^82,84^Rb. The nuclei turn out to be very soft with respect to γ deformation with a tendency to positive values in ^82^Rb but negative values in ^84^Rb. The values of γ = 20° and γ = 10° are used for ^82^Rb and ^84^Rb, respectively. The experimental and calculated *B*(M1)/*B*(E2) ratios are compared in Fig. 2. The experimental values in ^82^Rb are well reproduced in the calculations. This is also the case for ^84^Rb up to *ħω* ≈ 0.7 MeV. The increase of the experimental values at higher frequency can not be described within the assumed 4*qp* configuration. It is probably due to a change to a 6*qp* configuration.

The M1 bands C and D in ^83^Rb are irregular. Moreover, the experimental *B*(M1)/*B*(E2) ratios of these bands shown in Fig. 2 display a pronounced staggering which is not compatible with regular shears bands. In contrast to the odd-odd nuclei, the breakup of a pair of neutrons is necessary in ^83^Rb to generate 3*qp* or 5*qp* configurations of the shears type. This may drive the nuclear shape to very small quadrupole deformation, which is incapable of establishing a stable shears mechanism.

Summarizing, we have observed M1 bands in ^82^Rb, ^83^Rb, and ^84^Rb for the first time. The *B*(M1)/*B*(E2) ratios are of the order 10 (μ_N_/eb)^2^ to 20 (μ_N_/eb)^2^ and *decrease* with the angular momentum. This is characteristic for Magnetic Rotation. Thus, first evidence of the predicted existence of this new mode near *A* = 80 has been provided. The M1 bands in the doubly odd nuclei ^82^Rb and ^84^Rb can be described in the TAC model on the basis of a 4*qp* shears configuration. In contrast, the M1 bands in the odd-even nucleus ^83^Rb are not regular. The difference may be caused by the breakup of a neutron pair driving the nucleus to substantially smaller deformation, which is incapable of sustaining the shears mechanism.
